# Effects of Four Extraction Methods on Structure and In Vitro Fermentation Characteristics of Soluble Dietary Fiber from Rape Bee Pollen

**DOI:** 10.3390/molecules28124800

**Published:** 2023-06-16

**Authors:** Hui Zheng, Yan Sun, Yiqiong Zeng, Tao Zheng, Fan Jia, Pan Xu, Yao Xu, Yuxin Cao, Kai He, Yong Yang

**Affiliations:** 1 College of Pharmacy, Hunan University of Chinese Medicine, Changsha 410000, China; huizheng0104@163.com (H.Z.);; 2 School of Pharmaceutical Science, Hunan University of Medicine, Huaihua 418000, China

**Keywords:** bee pollen, soluble dietary fiber, extraction methods, structure, in vitro fermentation characteristics

## Abstract

In this study, soluble dietary fibers (SDFs) were extracted from rape bee pollen using four methods including acid extraction (AC), alkali extraction (AL), cellulase extraction (CL) and complex enzyme extraction (CE). The effects of different extraction methods on the structure of SDFs and in vitro fermentation characteristics were further investigated. The results showed that the four extraction methods significantly affected the monosaccharide composition molar ratio, molecular weight, surface microstructure and phenolic compounds content, but showed little effect on the typical functional groups and crystal structure. In addition, all SDFs decreased the *Firmicutes*/*Bacteroidota* ratio, promoted the growth of beneficial bacteria such as *Bacteroides*, *Parabacteroides* and *Phascolarctobacterium*, inhibited the growth of pathogenic bacteria such as *Escherichia-Shigella*, and increased the total short-chain fatty acids (SCFAs) concentrations by 1.63–2.45 times, suggesting that the bee pollen SDFs had a positive regulation on gut microbiota. Notably, the SDF obtained by CE exhibited the largest molecular weight, a relatively loose structure, higher extraction yield and phenolic compounds content and the highest SCFA concentration. Overall, our results indicated that CE was an appropriate extraction method of high-quality bee pollen SDF.

## 1. Introduction

Dietary fibers mean carbohydrate polymers with three or more monomeric units which are resistant to the endogenous digestive enzymes and thus neither hydrolyzed nor absorbed in the small intestine of humans [1]. Dietary fibers have exhibited many health benefits, such as cholesterol lowering, weight regulation, cancer prevention and regulating intestinal flora [2]. Based on the ability to be fully dispersed when mixed with water, dietary fibers can be classified as soluble and insoluble [3]. In contrast to the fecal bulking effect of most insoluble dietary fibers, most soluble dietary fibers (SDFs) can regulate gut microbiota and increase their metabolites such as short-chain fatty acids (SCFAs) to exert beneficial effects [1]. Due to good solubility and consumers’ demand for high dietary fiber healthy food, SDFs have become an ideal raw material for healthy food and are widely applied in various functional food industry. Plant-derived SDFs can be easily obtained from agricultural by-products such as coffee peel [4], pear fruit pomace [5], black soybean hulls [6] and plant raw materials with medicinal value such as ginseng [7], jujube (*Ziziphus jujuba* Mill.) [8] and sanchi (*Panax notoginseng*) flower [9], which have attracted the attention of many researchers due to their wide sources and potentially healthy functions. It has been noticed that extraction methods could affect the sugar chain breaking of carbohydrates in raw materials, resulting in a different structure and composition, which further influence the physicochemical and functional properties of SDFs [9].

Pollen is the male reproductive cells of flowers. Bee pollen is a fine, powder-like material made from pollen collected by bees from plant stamens mixed with nectar and bee secretions [10]. Bee pollen, known as a natural super food, has been used for medicinal and nutritional products in many countries. Historically, the food value of bee pollen has been restricted due to certain reasons such as low yield, its unique taste, allergic reactions and impurities such as fine sand, broken plant leaves and petals accidentally wrapped in by bees [11]. Therefore, early studies on bee pollen have mostly focused on the biological aspects of bees. In recent years, with the wide application of bee pollen collection equipment, the production of bee pollen sharply increased, thereby promoting the studies on the food value and healthy functions of bee pollen. Studies have shown that bee pollen has many biological activities on human health, such as antioxidant activity, antimicrobial activity, anti-inflammatory activity, anticarcinogenic activity and cardioprotective effects [12], and demonstrated that some healthy functions are closely related to regulating gut microbiota by bee pollen [11]. These studies mainly focused on phenolics and flavonoids in bee pollen [13], while there are have been few studies on the dietary fiber of bee pollen.

Our previous study on the analysis of fiber compositions of four kinds of bee pollens showed that the contents of bee pollen SDFs can reach 4–9% [14], which are rich compared to many cereals and pulses [15]. We also explored the extraction methods of bee pollen SDF by acid extraction, alkali extraction and cellulase extraction. Allergens of bee pollen are generally proteins [11], and enzyme-treated bee pollen can be used for allergens degradation into small peptides and amino acids resulting in decreased bee pollen allergenicity [16]. Therefore, the extraction process of bee pollen SDF is beneficial to remove the proteins associated with allergies and impurities in bee pollen, improving the food value of bee pollen and offering a new idea for the development of bee pollen products. At present, the studied plant-derived SDFs are mainly extracted from the flower, leaf, stem, fruit and seed cortex of plants, and there are almost no reports on SDF extraction from pollen. In this study, we used acid extraction, alkali extraction, cellulase extraction and complex enzyme extraction to extract SDFs from rape bee pollen, denoted as ACSDF, ALSDF, CLSDF and CESDF, respectively, and compared the effects of different extraction methods on the structure and regulating gut microbiota in vitro fermentation. This study could provide an appropriate extraction method of high-quality bee pollen SDF.

## 2. Results and Discussion

### 2.1. The Extraction Yield, Monosaccharide Composition and Molecular Weight of Bee Pollen SDFs

The extraction yield, monosaccharide composition and molecular weight of bee pollen SDFs are shown in Table 1. The extraction yield of CLSDF extracted by cellulase was the highest (6.01%), which was consistent with previous studies that cellulase contributed to improving the extraction yield of SDF [17]. The extraction yields of ACSDF and ALSDF were relatively low, which were 60.07% and 66.22% of CLSDF, respectively. The result may be due to their low molecular weight resulting in some polysaccharides not being effectively precipitated in ethanol or centrifuged. As shown in Table 1, the main monosaccharides in bee pollen SDFs were Arabinose, Galactose, Glucose, Xylose and Mannose, existing with different molar ratios in ACSDF (33.83%, 16.35%, 32.16%, 6.49%, 6.90%), ALSDF (40.99%, 20.07%, 13.53%, 13.75%, 4.83%), CLSDF (33.06%, 17.44%, 22.57%, 2.38%, 20.48%) and CESDF (29.98%, 15.08%, 17.79%, 8.37%, 19.00%), respectively. The results suggested that the main monosaccharide types of four extraction methods are similar, but different extraction methods significantly affected the molar ratio of monosaccharides in bee pollen SDF. This result is also similar with the previous studies [18]. The average molecular weights (Mw) of ACSDF, ALSDF, CLSDF and CESDF were 77.17, 56.50, 143.51 and 1050.27 kDa, respectively, which indicated that the four extraction methods had significantly effects on the molecular weight of bee pollen SDF. The Mw of CESDF was the largest, which might be because, compared with other methods, the reaction condition of CE was more gentle, and this is beneficial to the hydrolysis of starch, cellulose, and protein in bee pollen, resulting in lower damage to the sugar chain of SDF. Compared with the CESDF, the Mw/Mn of ACSDF, ALSDF and CLSDF were significantly smaller, suggesting that their molecular weight distributions were relatively concentrated and uniform.

### 2.2. Scanning Electron Microscopy (SEM)

**Figure 1:**
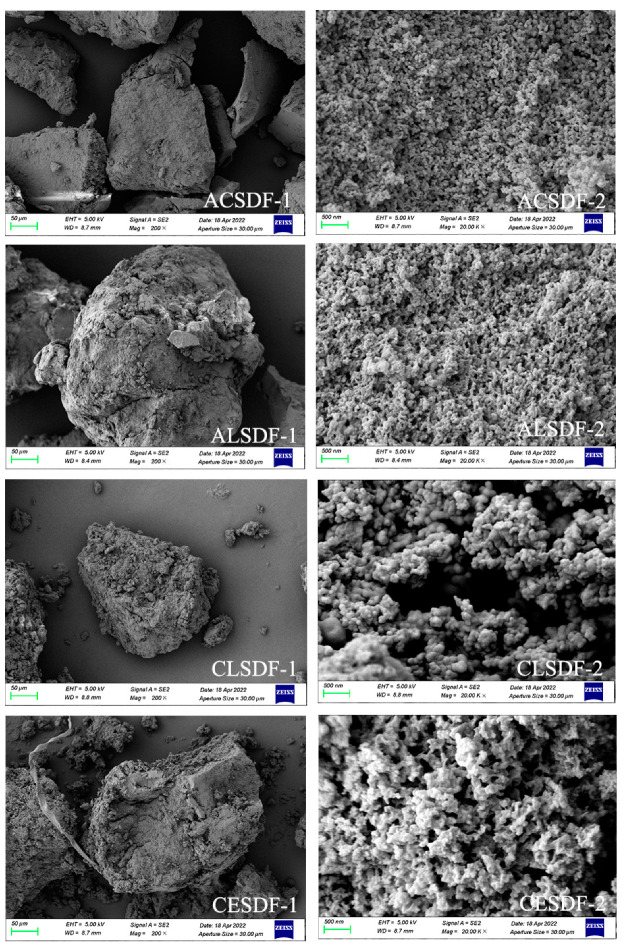
SEM images of ACSDF, ALSDF, CLSDF and CESDF.

SEM has become one of the most effective techniques to observe the microstructure of SDFs. The surface morphologies and microstructure of SDFs, such as particle size, network structure and surface holes, can affect the physicochemical and functional properties of SDF [19]. SEM images of ACSDF, ALSDF, CLSDF and CESDF are shown in Figure 1. At 200× magnification, ACSDF had fewer small particles scattered around and a smoother surface than those of the other SDFs. This was consistent with our previous study [20], which might indicate that ACSDF had better viscosity and was more compact than that of the other three SDFs.

At 20k× magnification, CLSDF showed the difference from the other three SDFs in surface morphologies. CLSDF was composed of irregularly small particles with relatively smooth surfaces, and the network structure between the particles was not obvious. ACSDF, ALSDF and CESDF exhibited an obvious network structure and numerous holes, which might be related to the cross-linkages between polysaccharide molecules during the process of ethanol precipitation. This result was similar to coffee peel SDF [4], pear fruit pomace SDF [5] and black soybean hulls SDF [6]. Compared with ACSDF and ALSDF, CESDF showed a rougher surface, and a looser and more obvious network structure, which may be due to the largest Mw of CESDF. This was similar to the studies of Wang et al. [19] and Wang et al. [21] on SDF extraction by different methods. ACSDF and ALSDF showed relatively compact network structures, which might be related to the hydrolysis of SDF into oligosaccharides with low molecular weight caused by acid or alkali during the extractions. 

### 2.3. Fourier Transform Infrared Spectroscopy (FT-IR)

**Figure 2:**
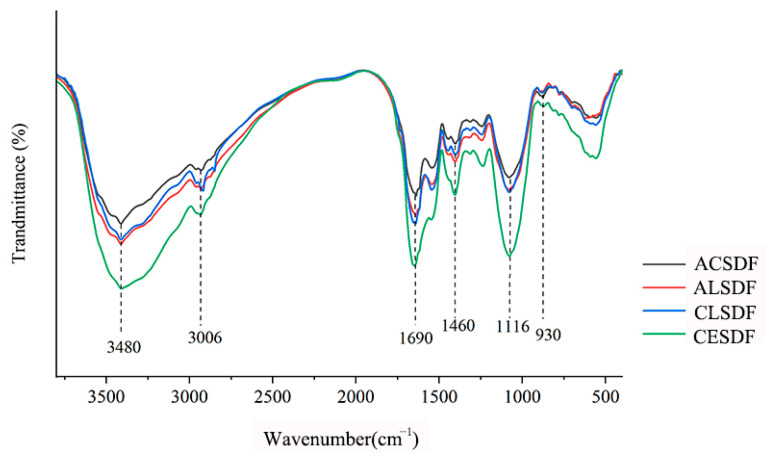
FT-IR spectra of ACSDF, ALSDF, CLSDF and CESDF.

FT-IR spectra of ACSDF, ALSDF, CLSDF and CESDF are shown in Figure 2, which exhibited the typical characteristics of polysaccharides, and were similar to tomato peels SDF [22], *Mesona chinensis* Benth residue SDF [23] and ginseng reside SDF [7]. In our study, SDFs had a stronger and wide peak intensity at 3480 cm^−1^, which was mostly attributed to O–H stretching vibration [18], and this might be relevant to free hydroxyl groups exposure in phenols and polysaccharides. The peak at 3006 cm^−1^ was assigned to the C–H stretching vibrations from the methyl and methylene groups [24]. The notable peak at 1690 cm^−1^ was assigned to the C=O stretching vibration of carboxylate groups, indicating that SDFs contain uronic acid [6]. The peak at 1460 cm^−1^ may be caused by the C–H group vibration [5]. The stronger absorption at 1116 cm^−1^ was assigned to the C–O stretching vibration and the O–H variable-angle vibration of C–O–C in the pyranose ring [6]. The peak at 930 cm^−1^ might indicate the characteristic absorption peak of β-pyranose [18].

FT-IR spectra of bee pollen SDFs had similar characteristic spectra and typical functional groups of polysaccharides, except that there were differences in the peak intensity of some absorption peaks. The peak intensity of those peaks from high to low in turn was roughly CESDF, ALSDF, CLSDF and ACSDF, which was similar to some previous studies on SDF extraction by different methods. This result of Wang et al. showed that the kiwifruit SDF by complex enzyme (α-amylase, protease and amyloglucosidase) extraction had a stronger peak intensity of some infrared absorption peaks than that by acid extraction [21]. In the studies of corn bran SDF [25] and black soybean hulls SDF [6], adding cellulase in SDF extraction contributed to the exposure of the functional groups and infrared peak intensity enhancement. The study of Jiang et al. indicated that sanchi (*Panax notoginseng*) flower SDF by α-amylase extraction had a stronger infrared peak intensity than that by acid and alkali solution extractions [9]. The differences in the FT-IR spectra of bee pollen SDFs may be mainly related to two factors: (1) chemical composition, which was related to the breakage modes of intermolecular and intramolecular chemical bonding during extraction [19]; and (2) particle size and microstructure [26]. As indicated by the SEM images, the relatively compact network structure of ACSDF might affect the exposure of functional groups, and the looser structure of CESDF might contribute to the exposure of functional groups.

### 2.4. X-ray Diffraction (XRD)

**Figure 3:**
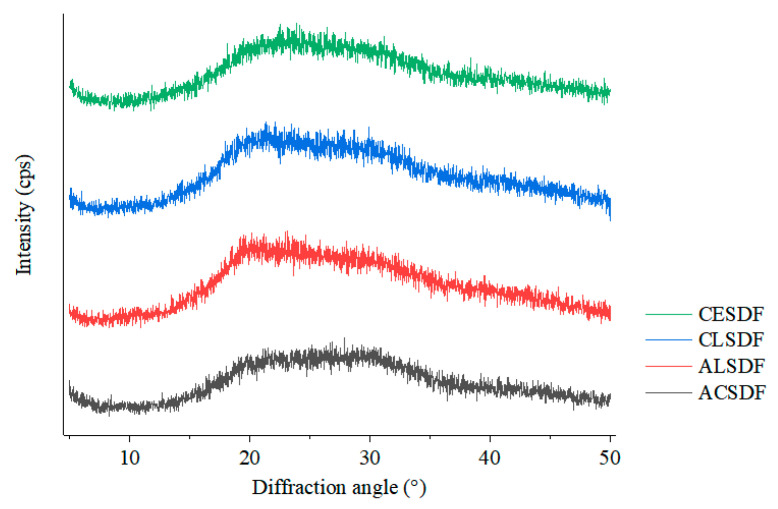
X-ray diffraction of ACSDF, ALSDF, CLSDF and CESDF.

X-ray diffraction is an efficient and convenient method to analyze the crystal structure of SDF. The X-ray diffractions of ACSDF, ALSDF, CLSDF and CESDF are shown in Figure 3. Bee pollen SDFs had similar characteristic spectra, indicating that the four extraction methods did not change the crystal structure of the bee pollen SDF. All bee pollen SDFs had the diffuse and wide peak intensity in the range of a 2θ diffraction angle from 20° to 30°, lacking sharp and strong diffraction peak, which suggested that bee pollen SDFs mainly exist in an almost amorphous state [27]. The X-ray diffraction of bee pollen SDFs did not show the obvious characteristics of cellulose I (the characteristic diffraction peaks appear at 2θ of 14.8°, 16.8° and 22.6°) and cellulose II (the characteristic diffraction peaks appear at 2θ of 12.1°, 19.8° and 22.0°) [27], which was different from many studies of SDF, such as *R. chingii* fruits [19], rice bran [28] and grapefruit peel [18]. This may be because the cell wall composition of pollen, the male reproductive cells of flowers, was different from that of other plant parts such as flower, leaf, stem, fruit and seed cortex.

### 2.5. Phenolic Compounds Determination of Bee Pollen SDFs

**Figure 4:**
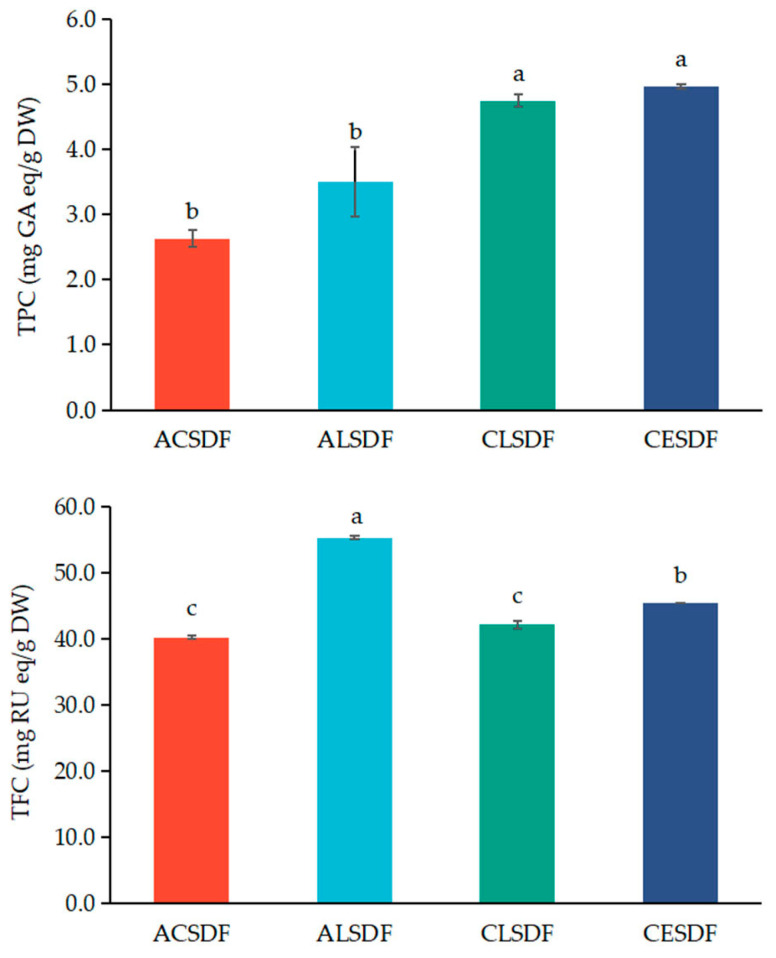
TPC and TFC of ACSDF, ALSDF, CLSDF and CESDF. The different superscript letters indicate a significant difference (*p* < 0.05) based on Duncan’s test.

Bee pollen contains significant amounts of phenolic compounds which have high antioxidant activity. Studies have confirmed that phenolic compounds in bee pollen are closely related to the biological activity of bee pollen such as anti-inflammatory properties, antibacterial effects, anticarcinogenic properties and immunostimulatory activity [13]. The contents of phenolic compounds have become one of the important indexes of bee pollen quality evaluation and process optimization [29]. Phenolic compounds in bee pollen mainly include flavonoids and phenolic acids [13], and their contents and compositions are closely associated with numerous factors, such as botanical origin, planting environment, climatic conditions, bee race and beekeeping activity [30]. Researchers have reported that SDF extracted from polyphenol-rich raw material contains phenolic compounds, which affect the functional properties of SDF [31]. The total polyphenol contents (TPCs) and total flavonoid contents (TFCs) in ACSDF, ALSDF, CLSDF and CESDF are shown in Figure 4. The TFCs in bee pollen SDFs were higher than the TPC contents in bee pollen SDFs, which were consistent with the results of Zhang et al. [29] which showed that the TFCs were higher than the TPCs in rape bee pollen. Compared with the TPC in ACSDF and ALSDF, the TPCs in CESDF and CLSDF were higher, and the TPC in CESDF was slightly higher than that in CLSDF, but there was no significant difference between them (*p* > 0.05). The TFC in ALSDF was higher than that in other three SDFs, which may be due to the easier dissolution of flavonoids in alkali solution. Overall, the TPC and TFC in CESDF were relatively higher. As indicated by the SEM images, CESDF exhibited the relatively loose structure, making it easier to encapsulate and intercept phenolic compounds, or bind phenolic compounds by covalent (ester bond) and non-covalent (strong hydrogen-bonding) interactions [32]. 

### 2.6. SCFA Concentration after In Vitro Fermentation

SCFAs are key bacterial metabolites that affect various physiological processes and may contribute to health and disease [33]. Studies have shown that dietary fiber in the colon is fermented by gut microbiota to produce SCFAs, mainly acetic acid, propionic acid and butyric acid [33]. Previous studies showed that the acetic acid, propionic acid, butyric acid and total SCFA concentrations in polysaccharides gradually increased as the vitro fermentation progressed [34]. In our study, SCFA concentrations were measured after 48 h in vitro fermentation in the blank group and SDF groups. The results are shown in Table 2. Compared with the blank group, the SDF group significantly increased SCFA concentrations after in vitro fermentation for 48 h. The total SCFA concentrations in SDF groups were 1.63–2.45 times of that of the blank group, and the total SCFA concentrations in CESDF group were the highest. Acetic acid is an important energy source for intestinal cells and the most abundant SCFA in peripheral circulation, and has positive effects on health such as obesity, skeletal muscle functioning and natural aging-related disorders [35,36]. In our study, the acetic acid concentration was the highest during in vitro fermentation, which was consistent with a previous study in which carbohydrates can produce the most acetate [37] and the acetic acid concentrations of ACSDF, ALSDF, CLSDF and CESDF were 1.57, 1.46, 1.55 and 2.43 times of that of the blank group, respectively. Propionic acid can be converted into glucose through gluconeogenesis, with anti-inflammatory, antioxidant and immunomodulatory mechanism properties [38,39]. The propionic acid concentrations of ACSDF, ALSDF, CLSDF and CESDF were 2.33, 2.45, 2.23 and 1.88 times that of the blank group, respectively. Although the propionic acid concentration of CESDF was relatively low, there was no significant difference compared to the other three SDFs. Studies have proven that butyric acid is the main energy source of colon cells, which can maintain decreased arterial blood pressure, intestinal homeostasis, as well as prevent intestinal inflammation and colorectal cancer [40,41]. The butyric acid concentrations of ACSDF, ALSDF, CLSDF and CESDF were 1.69, 1.93, 2.22 and 2.91 times of that of the blank group, respectively. Studies have shown that SCFAs are positively correlated with *Bacteroidetes,* which are believed to produce acetic acid and propionic acid through fermentation [42], which was consistent with our result. In general, bee pollen SDFs significantly increased the concentration of SCFAs compared with the blank, especially the CESDF, suggesting that SDFs may play health functions by regulating gut microbiota to produce SCFAs.

### 2.7. Effects of Bee Pollen SDFs In Vitro Fermentation on the Gut Microbiota

**Figure 5:**
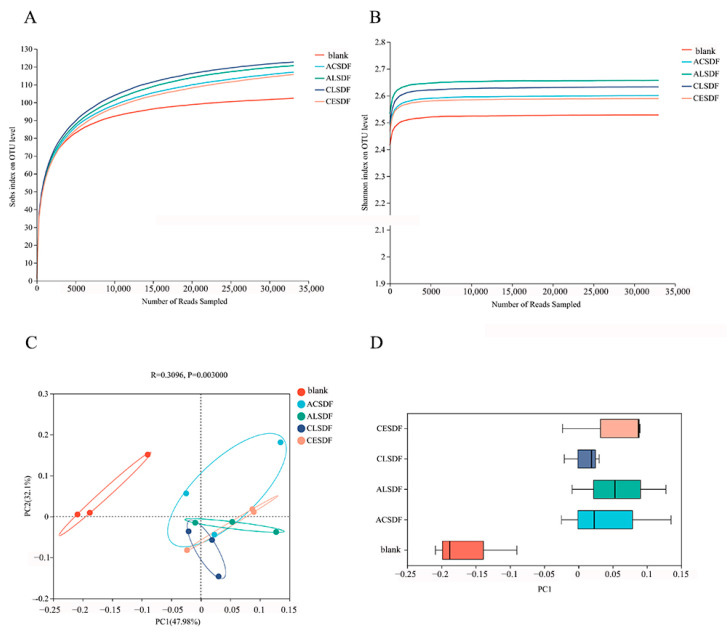
Rarefaction curves (**A**), Shannon curves (**B**), principal coordinates analysis (PCoA) (**C**) and discrete distribution on PC1 (**D**) of gut microbiota.

In order to explore the effects of the bee pollen SDFs on gut microbiota composition, the analyses of gut microbiota were performed after in vitro simulated saliva-gastrointestinal digestion and 48 h fermentation. Additionally, for identifying the statistically significant difference in the regulation on gut microbiota between the blank and bee pollen SDF, ACSDF, ALSDF, CLSDF and CESDF were combined as the SDF group for analysis in some of the results. The rarefaction curves and Shannon curves of samples are shown in Figure 5A,B, which tended to be flat with the increase in the number of reads sampled, indicating that the sequencing depth and data volume were reasonable, and can represent the diversity and richness of gut microbiota. 

The principal co-ordinates analysis (PCoA) in Figure 5C exhibited that PC1 and PC2 contributed 47.98% and 32.10% of the variation in gut microbiota among the 100 most dominant OTUs across all substrates, respectively. Combined with the discrete distribution on PC1 shown in Figure 5D, the distance between the blank group and the bee pollen SDF group was far away, and there was statistical separation, indicating low similarity between the blank group and SDF group. Compared with the blank, bee pollen SDFs played a significant role in the regulation of gut microbiota.

**Figure 6:**
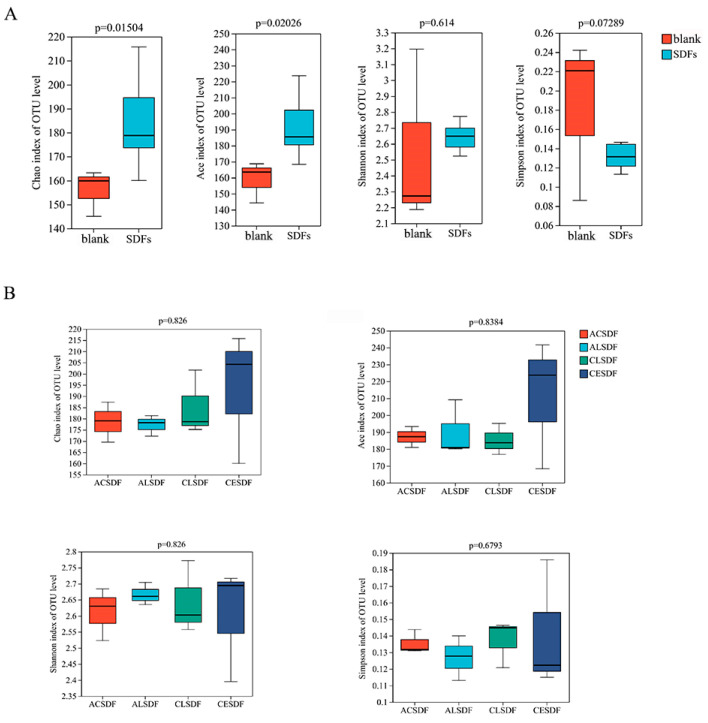
Alpha diversity of gut microbiota between the blank group and bee pollen SDF group (**A**) and among SDF groups (**B**).

The indicators of Chao, Ace, Shannon and Simpson in Alpha diversity are shown in Figure 6. Chao and Ace mainly reflect the richness of microbial community, and Shannon and Simpson mainly exhibit the diversity of community distribution. Compared with the blank group, Chao and Ace indicators of bee pollen SDF group were significantly increased. The Shannon and Simpson indicators of the bee pollen SDF group were also different from those in the blank group, but there was no significant difference. The results suggested that the bee pollen SDF group significantly modulated the richness of microbial community, which was consistent with previous studies of SDFs [1]. In addition, the Alpha diversity results of the microbial community among SDF groups are shown in Figure 6B. Chao, Ace and Shannon indicators of CESDF were relatively higher than those of other SDFs, suggesting that CE extraction may be more conducive to improving the richness and diversity of the microbial community.

**Figure 7:**
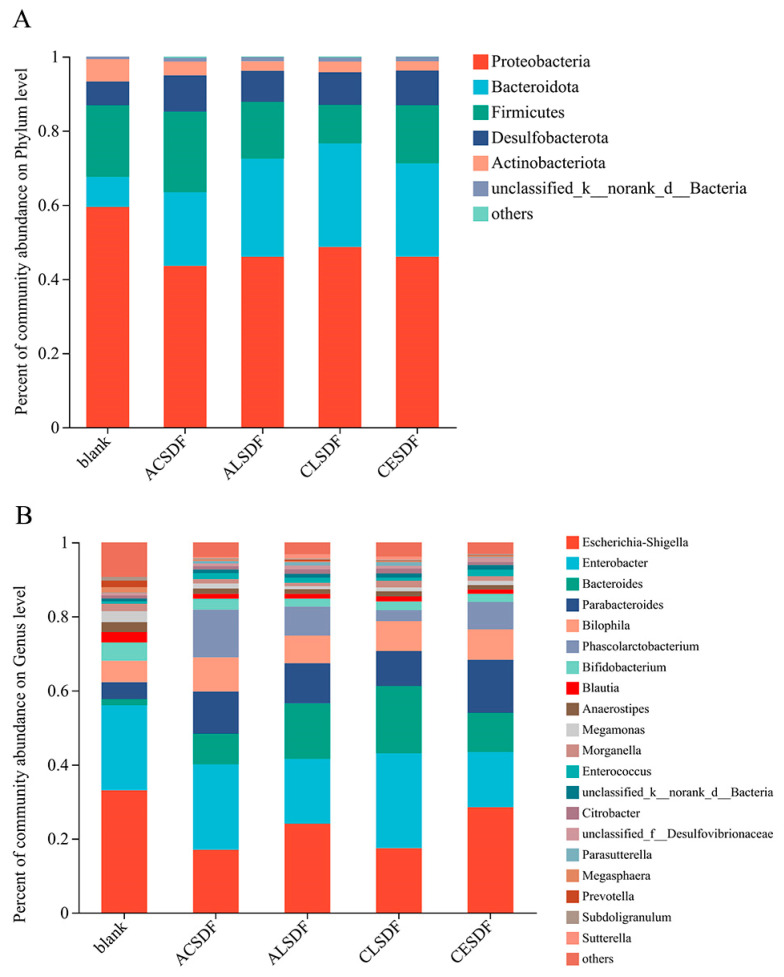
The relative abundance percent of gut microbiota at the phylum (**A**) and genus (**B**) levels.

The result of the relative abundance percentage of gut microbiota on the phylum level is shown in Figure 7A. The dominant phylum of human fecal microbiota in vitro fermentation was mainly composed of *Proteobacteria*, *Bacteroidota*, *Firmicutes*, *Desulfobacterota* and *Actinobacteriota*, which was similar to some previously reported studies [43]. On the whole, compared with the blank group, the bee pollen SDF group contributed to increasing the abundance of *Bacteroidota* and *Desulfobacterota*, and decreasing the abundance of *Proteobacteria* and *Actinobacteriota*. *Bacteroidota* isolated from human feces was considered a major degrader of fiber and polysaccharide components due to the carbohydrate-degrading enzymes contained in gene clusters [44]. As a result, there was a greater increase in the abundance of *Bacteroidetes* in the bee pollen SDF group. Moreover, the *Firmicutes*/*Bacteroidota* ratios (F/B) of the ACSDF, ALSDF, CLSDF and CESDF groups were 1.09, 0.58, 0.37 and 0.63, respectively, which were absolutely lower than that of the blank group (2.39). Some studies have demonstrated that a low F/B is negatively correlated with obesity [45], so the result might indicate that the bee pollen SDFs have a weight loss function by regulating gut microbiota, similar to previous studies [45]. The abundance of *Proteobacteria* in the bee pollen SDF group (43.62–48.46%) was lower than that of the blank group (59.40%). *Proteobacteria* can be used as a potential diagnostic signature of dysbiosis and risk of disease [46], therefore the result suggested that the bee pollen SDFs can improve the function of gut microbiota. The abundance of *Desulfobacterota* in the bee pollen SDF group increased compared with the blank group, which was similar to the study of extracellular polysaccharides from *Sporidiobolus pararoseus* on gut microbiota [47]. 

The result of the relative abundance percent of gut microbiota at the genus level is shown in Figure 7B. *Escherichia-Shigella*, which is commonly regarded as an important pathobiont [48], was a dominant genus of gut microbiota after in vitro fermentation in our study, covering 16.96–36.29% of the abundance in all groups. Compared with the blank group, the abundance of *Escherichia-Shigella* greatly decreased in the bee pollen SDF group. *Bacteroides* and *Parabacteroides* as two important genus of *Bacteroidetes* [49], were richer in the bee pollen SDF group, and the results were consistent with the characteristic of polysaccharide degradation by *Bacteroidetes*. The abundances of *Bacteroides* and *Parabacteroides* covering 8.28–18.22% and 9.43–14.35% of the abundance in the bee pollen SDF group, respectively, were significantly higher than those in the blank group, by 1.68 and 4.55%, respectively. Previous studies have displayed that *Parabacteroides* has protective effects on metabolic syndrome, inflammatory bowel disease and obesity [50]. *Phascolarctobacterium,* which was reported to be positively associated with positive emotions in humans [51], mainly uses succinate produced by other bacteria for growth [52]. Thus, the increased abundance of *Bacteroides* and *Paracbacteroides*, the main producers of succinic acid, resulted in an abundance of *Phascolarctobacterium* in the bee pollen SDF group, and almost no *Phascolarctobacterium* in the blank group. Compared with the blank group, the abundance of *Bilophila* in the bee pollen SDF group increased. *Bilophila* was reported to be associated with the gut dysbiosis of pancolitis in patients with ulcerative colitis [53], which is also commonly found in the gut flora of healthy humans and may alleviate cardiovascular disease in the host [54]. *Blautia* and *Bifidobacterium* were wildly reported to have probiotic characteristics, such as biological transformation, the regulation of host health and metabolic syndrome alleviation [55,56]. Compared with the blank group, the abundances of *Blautia* and *Bifidobacterium* decreased in the bee pollen SDF group, which may be related to changes in the abundance of *Firmicutes* and *Actinobacteriota* in our study.

**Figure 8:**
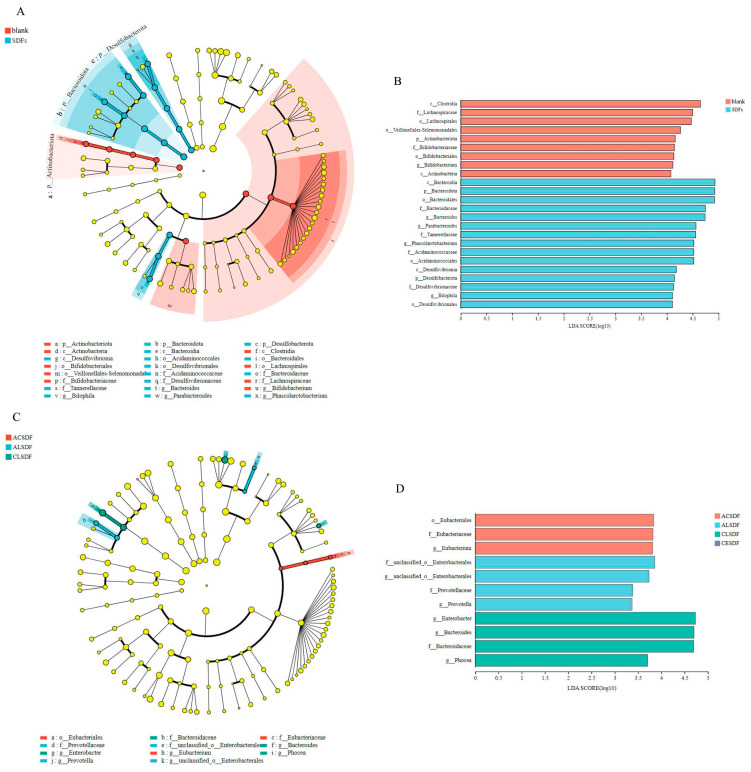
LEfSe (**A**) and LDA (**B**) of the microbiota composition between the blank group and bee pollen SDF group, LEfSe (**C**) and LDA (**D**) of the microbiota composition among the SDF groups.

The linear discriminant analysis effect size (LEfSe) among the blank group and bee pollen SDF group is shown in Figure 8A, and the linear discriminant analysis to estimate the impact of each biomarker (LDA) (score > 4, *p* < 0.05) is shown in Figure 8B. There were 24 OTUs that were significantly different between the blank group and the bee pollen SDF group. The statistically significant biomarkers in the blank group mainly were the *Lachnospiraceae* and *Bifidobacteriaceae* families and their generations. It has been reported that metabolic syndrome, obesity, diabetes, liver diseases, inflammatory bowel disease and chronic kidney disease are associated with inflammatory conditions involving the *Lachnospiraceae* [57]. The statistically significant biomarkers in the bee pollen SDF group were mainly the *Bacteroidaceae*, *Tannerellaceae, Acidaminococcaceae* and *Desulfovibrionaceae* families and their generations. The LEfSe among the four bee pollen SDF groups is shown in Figure 8C, and LDA (score > 3, *p* < 0.05) is shown in Figure 8D. There were 11 OTUs that were significantly different between the four bee pollen SDF groups. The statistically significant biomarkers in the ACSDF, ALSDF and CLSDF groups were mainly *Eubacteriaceae*, *Prevotellaceae* and *Bacteroidaceae* families and their generations, respectively. 

In our study, bee pollen SDFs significantly modulated the composition of gut microbiota in vitro fermentation. Compared with the blank group, there was a higher relative abundance of *Bacteroidota*, lower F/B and lower relative abundances of *Proteobacteria*, especially *Escherichia-Shigella* in the bee pollen SDF group. It was suggested that the bee pollen SDFs could decrease the abundances of harmful intestinal microbiota and positively regulate intestinal microbiota, while the LEfSe analysis among the four bee pollen SDF groups showed that the effects of different extraction methods on gut microbiota were less different. The previous study reported that the effects of SDFs on gut microbiota vary widely depending on the type of fiber, crystalline form and the degree of polymerization [58]. In our study, the types of the four SDFs can be considered roughly similar because they come from the same raw material, and there was little difference in the crystal structure, which may explain the small difference on regulation gut microbiota. Among them, the CESDF group exhibited the higher microbial richness and diversity of the microbial community, which may explain the higher SCFA concentration in CESDF group than in the other three SDF groups. This result may also be related to the largest molecular weight, relatively loose structure and higher phenolic compounds content of CESDF, which requires further study.

## 3. Materials and Methods

### 3.1. Materials

Rape bee pollen from Hubei, China, was provided by Changsha Bee Dance Human Biotechnology Co., Ltd. (Changsha, Hunan, China). Cellulase (CAS 9012-54-8, S10041) was bought from Shanghai Yuanye Biotechnology Co., Ltd. (Shanghai, China). Acetic acid, propionic acid and butyric acid were bought from Shanghai Macklin Biochemical Technology Co., Ltd. (Shanghai, China) and were analytically pure. Total dietary fiber assay kit (TDF-200A) was bought from Megazyme International Ireland Ltd. (Bray, Ireland). Lipase, α-amylase, pepsin, trypsin and bile acid were bought from Shanghai Bioengineering Co., Ltd. (Shanghai, China) and were analytically pure. Rutin and gallic acid standards, 98% purity, were bought from Chengdu Aifa Biotechnology Co., Ltd. (Chengdu, Sichuan, China). The remaining reagents were bought from Sinopharm Chemical Reagent Co., Ltd. (Shanghai, China) and were analytically pure.

### 3.2. Extractions of Bee Pollen Soluble Dietary Fiber

#### 3.2.1. Acid Extraction

The method of acid extraction was based on the methods of our previous study [20] and Gan et al. [18] with some modifications. Bee pollen (100 g) was mixed with 1500 mL of hydrochloric acid (pH 4.3). The mixture was incubated at 50 °C, 250 rpm for 2 h with vibration and centrifugated at 5000 rpm for 20 min to collect the supernatant. The supernatant was concentrated down to one third of its original volume under reduced pressure in a rotary evaporator at 60 °C, and pH was adjusted to 7.0. Next, the supernatant was mixed with four times the volumes of 95% ethanol, which was placed for 12 h at 5 °C. Then, centrifuged at 5000 rpm for 15 min to collect the precipitate, which was dissolved in distilled water to remove the ethanol by rotary evaporation. The acid extracted bee pollen soluble dietary fiber (ACSDF) was obtained by freeze-drying. The extraction yield (%) of bee pollen soluble dietary fiber was calculated according to Wang et al. [21].

(1) $$yield\;(\%)=\frac{C}{W}\times 100\%$$

where C is the weight of bee pollen soluble dietary fiber (g) and W is the weight of bee pollen (g).

#### 3.2.2. Alkali Extraction

The method of alkali extraction was based on the methods of Gan et al. [18] with some modifications. Bee pollen (100 g) was mixed with 1500 mL of 0.1% (*w*/*v*) NaOH solution. The mixture was incubated at 50 °C, 250 rpm for 2 h with vibration and was centrifugated at 5000 rpm for 20 min to collect the supernatant. The rest of the extraction process was as detailed in Section 3.2.1. The alkali-extracted bee pollen soluble dietary fiber (ALSDF) was obtained.

#### 3.2.3. Cellulase Extraction

The method of cellulase extraction was based on the methods of our previous study [20] and Gan et al. [18] with some modifications. Bee pollen (100 g) and cellulase (2.5 g) were mixed with 1500 mL of hydrochloric acid (pH 4.0). The mixture was incubated at 50 °C, with 250 rpm for 2 h with vibration, heated at 85 °C for 10 min to inactivate the cellulase activity, and cooled to room temperature. After that, the mixture was centrifugated at 5000 rpm for 20 min to collect the supernatant. The rest of the extraction process was performed as detailed in Section 3.2.1. The cellulase-extracted bee pollen soluble dietary fiber (CLSDF) was obtained.

#### 3.2.4. Complex Enzyme Extraction

The method of complex enzyme extraction using a total dietary fiber assay kit was slightly modified according to Gu et al. [59]. Bee pollen (10 g) was mixed with 400 mL of MES-TRIS buffer (pH 8.3) and fully stirred. Then, 500 µL of α-amylase solution was added, and the mixture was hydrolyzed in 98 °C water bath for 30 min. Next, 1 mL of protease solution was added, and the mixture was hydrolyzed in a 60 °C water bath for 30 min. After that, 2 mL of amyloglucosidase solution was added after adjusting the pH to 7.0, and the sample was hydrolyzed in a 60 °C water bath for 30 min. The rest of the extraction process was performed as detailed in Section 3.2.1. The complex enzyme-extracted bee pollen soluble dietary fiber (CESDF) was obtained.

### 3.3. Monosaccharide Composition Analysis

Monosaccharide composition of bee pollen SDF was determined as described by Ma et al. [60]. Bee pollen (5 mg) was hydrolyzed with 1 mL of 2 M trifluoroacetic acid at 121 °C for 2 h in a sealed tube, and dried with nitrogen. Add methanol to wash and then blow dry, repeat 3 times. The residue was re-dissolved in deionized water and filtered through 0.22 μm microporous filtering film for measurement. The sample was analyzed by high-performance anion-exchange chromatography on a CarboPac PA-20 anion-exchange column (150 mm × 3.0 mm, 10 μm; Dionex, Sunnyvale, CA, USA) using a pulsed amperometric detector (PAD; Dionex ICS 5000+ system). Flow rate, 0.5 mL/min; injection volume, 5 μL; solvent system A: (ddH_2_O), solvent system B: (0.1 M NaOH), solvent system C: (0.1 M NaOH, 0.2 M NaAc); gradient program, volume ratio of solution A, B and C was 95:5:0 at 0 min, 85:5:10 at 26 min, 85:5:10 at 42 min, 60:0:40 at 42.1 min, 60:40:0 at 52 min, 95:5:0 at 52.1 min, and 95:5:0 at 60 min. The standard monosaccharides were also analyzed in the same way.

### 3.4. Molecular Weight Analysis

The sample was dissolved in an 0.1 M NaNO_3_ aqueous solution at the concentration of 1 mg/mL, and filtered through a 0.45 μm microporous filtering film for measurement. The weight-average molecular weight (Mw) and polydispersity (Mw/Mn) of bee pollen SDF were determined as described by Lin et al. using SEC-MALLS-RI [61]. 

### 3.5. SEM

The electron microscopy observation and photographing of bee pollen SDF were analyzed with an SEM (EVO18, Carl Zeiss AG, Jena, Germany). The sample was loaded on a sample holder with double-sided conducting adhesive tapes, and coated with a gold layer. Subsequently, the sample was observed at 200× and 20k× magnification at 5.0 kV.

### 3.6. FT-IR

The FTIR spectroscopy instrument (Nicolet 380, Thermo Fisher Scientific Inc., Waltham, MA, USA) was used for the FTIR spectra of bee pollen SDF, and the absorption was recorded in the wavelength from 4000 to 400 cm^−1^ [21]. Sample (2 mg) was mixed with 200 mg of KBr, followed by pressing into one slice, and the mixture was scanned for 32 times at a resolution of 4 cm^−1^.

### 3.7. XRD

The XRD analysis of bee pollen SDF was measured by an X-ray diffractometer (D8 Advance, Bruker AXS Co., Ltd., Karlsruhe, Germany). The determination was performed at room temperature using a Cu-Kα radiation source with a step size of 0.02°. The diffraction angle (2θ) was performed from 5 to 50° with a speed of 1°/min.

### 3.8. Phenolic Compounds’ Determination

#### 3.8.1. Extraction of Phenolic Compounds

The method was based on our previous study [62] with some modifications. Bee pollen SDF (0.5 g) was mixed with 10 mL of 70% ethanol and ultrasonicated at 60 °C for 50 min, before being centrifugated at 5000 rpm for 15 min. After repeat extraction, the supernatants were collected and combined, and diluted with 70% ethanol to 50 mL. The phenolic extract solution was obtained.

#### 3.8.2. TPCs Determination

The method was based on our previous study [63]. The extract solution (100 μL) was mixed with 7.9 mL of distilled water and 500 μL of Folin–Ciocalteu reagent, then after 5 min, 1.5 mL of 20% (*w*/*v*) sodium carbonate solution was added. After resting for 2 h at room temperature in the dark, the absorbance at 765 nm was measured using a spectrophotometer (UV-1780, Suzhou Shimadzu Instrument Co., Ltd., Suzhou, Jiangsu, China). The result was expressed as a mg of gallic acid (GA) equivalent in 1 g dry weight of sample (mg GA eq/g DW).

#### 3.8.3. TFCs Determination

Total flavonoid contents of SDFs were determined using the NaNO_2_-Al(NO_3_)_3_ method [64]. The extract solution (200 μL) was mixed with 400 μL of 5% (*w*/*v*) NaNO_2_ solution, and was left to stand in the dark at room temperature for 6 min. Next, the mixture was added with 400 μL of 10% (*w*/*v*) Al(NO_3_)_3_ solution and left to stand for 6 min. After that, 4 mL of 4% (*w*/*v*) NaOH solution and 5 mL of distilled water were added, and then left to stand for 15 min. The absorbance at 510 nm was measured using a spectrophotometer (UV-1780, Suzhou Shimadzu Instrument Co., Ltd., Suzhou, Jiangsu, China). The result was expressed as a mg of rutin (RU) equivalent in 1 g dry weight of the sample (mg RU eq/g DW).

### 3.9. In Vitro Simulated Saliva-Gastrointestinal Digestion of Bee Pollen SDF

The in vitro simulated saliva–gastrointestinal digestion experiments of bee pollen SDF, including sequential oral, gastric and intestinal digestion, were performed according to Brodkorb et al. [65]. The solution of the bee pollen SDF after in vitro simulating the digestion was vacuum-concentrated and freeze-dried to obtain the digested residue of bee pollen SDF. Additionally, the ultra-pure water added to the simulated digestion medium was used as the blank group.

### 3.10. In Vitro Fermentation of Bee Pollen SDF

The growth medium of gut microbiota was carried out as described by Amorim et al. [66]: peptone water (2 g/L), yeast extract (2 g/L), NaCl (0.1 g/L), KH_2_PO_4_ (40 mg/L), K_2_HPO_4_ (40 mg/L), MgSO_4_·7H_2_O (0.01 g/L), NaHCO_3_ (2 g/L), CaCl_2_·6H_2_O (0.01 g/L), Tween 80 (14.8 mL/L), bile salts (0.5 g/L), hemin (5 mg/L), cysteine HCl (0.5 g/L), vitamin K_1_ (74.1 μL/L), Na_2_S·9H_2_O (0.8 mmol/ L) and resazurine (1 mg/L).

The human fecal microbiota and in vitro fermentation experiments were carried out as described by Liu et al. [67], with slight modifications. The feces of three healthy volunteers (two males and one female, with mean body mass index = 20.3 and a mean age = 20.6 years) which did not take antibiotics within three months were collected. After discarding both ends and the surface of the feces, 10 g feces was added into 100 mL sterile phosphate-buffered solution and efficiently stirred. The human fecal slurry was obtained after filtering with 4 layers of gauze, then moved into C-11 Mitsubishi anaerobic gas bag (Mitsubishi Chemical Co., Tokyo, Japan) by using C-22 (Mitsubishi Chemical Co.) as an anaerobic indicator, and used as soon as possible. The digested residue (100 mg) and 9 mL of the growth medium were mixed in the 20 mL centrifuge tube, and autoclaved at 120 °C for 20 min, and then cooled down to room temperature. Next, 1 mL of the human fecal slurry was added to the mixed solution and anaerobically fermented by moved into C-11 Mitsubishi anaerobic gas cylinder by using C-22 as an anaerobic indicator at 50 °C, 100 rpm for 48 h with vibration and centrifuged at 11,000 r/min for 10 min. The supernatant was collected and stored at −60 °C for SCFA analysis, and the sediment was stored at −60 °C for gut microbiota analysis.

### 3.11. The SCFA Analysis

The method was slightly modified according to Yang et al. [68]. The SCFA concentrations, including acetic acid, propionic acid and butyric acid, after 48 h in vitro fermentation of SDFs by human fecal microbiota were determined by HPLC (1260, Agilent Technologies Co. Ltd., Santa Clara, CA, USA) with a reversal phase column (XB-C18, Agilent Technologies Co. Ltd.). The supernatants after the in vitro fermentation of SDFs were thawed and filtered through a 0.45 µm microporous membrane before injection. HPLC conditions: column temperature 30 °C, detection wavelength 210 nm, injection volume 10 µL, flow rate 1.0 mL/min. The mobile phases were 95% (*w*/*v*) phosphoric acid solution (pH 2.8) and 5% methanol.

### 3.12. The Gut Microbiota Analysis

The total microbial genomic DNA was extracted from sediment after 48 h of in vitro fermentation using the E.Z.N.A.^®^ soil DNA Kit (Omega Bio-tek, Norcross, GA, USA), and the quality and concentration of DNA were checked by 1.0% agarose gel electrophoresis and a NanoDrop^®^ ND-2000 spectrophotometer (Thermo Scientific Inc., Waltham, MA, USA). The hypervariable region V3–V4 of the bacterial 16S rRNA gene was amplified with primer pairs 338F (5′-ACTCCTACGGGAGGCAGCAG-3′) and 806R (5′-GGACTACHVGGGTWTCTAAT-3′). Additionally, the amplified products were sequenced on an Illumina MiSeq PE300 platform (Illumina, San Diego, CA, USA) according to the standard protocols by Majorbio Bio-Pharm Technology Co. Ltd. (Shanghai, China). The data were analyzed on the online platform of Majorbio Cloud Platform (www.majorbio.com).

### 3.13. Statistic Analysis

Samples were prepared and analyzed in triplicate. All data are expressed as the means ± standard deviation (SD). Multiple group comparison was undertaken by ANOVA and Duncan multiple tests using IBM SPSS version 23 (IBM SPSS, Armonk, NY, USA) (*p* < 0.05).

## 4. Conclusions

In this study, bee pollen SDFs were extracted by four methods including AC, AL, CL and CE. Among them, SDF extracted by CE showed the largest molecular weight (Mw: 1050.27 kDa), relatively loose structure and higher phenolic compounds content. Moreover, after incubation with human fecal microbiota, SDF extracted by CE increased the microbial richness and diversity of the microbial community. Additionally, the SCFA concentration in the CESDF group was the highest among the four groups. These results indicated that the CE was an appropriate extraction method of high-quality bee pollen SDF. Our findings will provide a theoretical foundation for the application of SDFs as a high-quality dietary fiber supplement.

## Figures and Tables

**Table 1:** The extraction yield, monosaccharide composition and molecular weight of bee pollen SDFs.

Treatment	ACSDF	ALSDF	CLSDF	CESDF
Extraction yield (%)	3.61	3.98	6.01	5.05
Monosaccharide composition (molar ratio %)				
Fucose	1.20	1.42	0.71	0.46
Arabinose	33.83	40.99	33.06	29.98
Rhamnose	0.82	3.56	0.69	3.45
Galactose	16.35	20.07	17.44	15.08
Glucose	32.16	13.53	22.57	17.79
Xylose	6.49	13.75	2.38	8.37
Mannose	6.90	4.83	20.48	19.00
Ribose	ND	ND	ND	3.47
Glucuronic acid	2.25	1.85	2.66	2.33
Molecular weight				
Mw (kDa)	77.17	56.50	143.51	1050.27
Mn (kDa)	20.91	15.27	32.23	18.75
D (Mw/Mn)	3.69	3.70	4.45	56.02

**Table 2:** SCFA concentration after in vitro fermentation 48 h of SDFs.

SCFAs (mmol/L)	Blank	ACSDF	ALSDF	CLSDF	CESDF
Acetic acid	3.81 ± 0.73 ^d^	6.00 ± 0.47 ^b^	5.57 ± 0.22 ^bc^	5.92 ± 0.55 ^b^	9.24 ± 1.79 ^a^
Propionic acid	0.48 ± 0.06 ^b^	1.12 ± 0.18 ^a^	1.18 ± 0.16 ^a^	1.07 ± 0.15 ^a^	0.90 ± 0.15 ^a^
Butyric acid	0.81 ± 0.07 ^d^	1.37 ± 0.05 ^c^	1.56 ± 0.17 ^c^	1.80 ± 0.05 ^b^	2.36 ± 0.10 ^a^
Total SCFAs	5.10 ± 0.68 ^c^	8.49 ± 0.32 ^b^	8.31 ± 0.20 ^b^	8.79 ± 0.44 ^b^	12.50 ± 1.76 ^a^

Values represent the mean ± SD (*n* = 3). The different superscript letters indicate a significant difference (*p* < 0.05) within the row based on Duncan’s test.

## Data Availability

Data are contained within the article.
